# Relationship of Chronic Stress and Hypertension with Bone Resorption

**DOI:** 10.3390/jfmk10010021

**Published:** 2025-01-04

**Authors:** Marina Ribeiro Paulini, Mariangeles Aimone, Sara Feldman, Daniela Vieira Buchaim, Rogerio Leone Buchaim, João Paulo Mardegan Issa

**Affiliations:** 1 Department of Basic and Oral Biology, School of Dentistry of Ribeirão Preto, University of São Paulo (FORP-USP), Ribeirão Preto 14040-904, Brazil; marina.paulini@usp.br; 2 LABOATEM, Laboratory of Ostearticular Biology and Tissue Engineering, School of Medicine, Rosario S2002, Argentina; mariangelessaimone@gmail.com (M.A.); saryfeldman@gmail.com (S.F.); 3 Research Council, National Rosario University (CIUNR-CONICET), Rosario S2002, Argentina; 4 Medical School, University Center of Adamantina (FAI), Adamantina 17800-000, Brazil; danibuchaim@alumni.usp.br; 5 Graduate Program in Anatomy of Domestic and Wild Animals, Faculty of Veterinary Medicine and Animal Science, University of São Paulo (FMVZ/USP), Sao Paulo 05508-270, Brazil; rogerio@fob.usp.br; 6 Center for the Study of Venoms and Venomous Animals, São Paulo State University (CEVAP/UNESP), Botucatu 18619-002, Brazil; 7 Department of Biological Sciences, School of Dentistry of Bauru, University of São Paulo (FOB-USP), Bauru 17012-901, Brazil

**Keywords:** hypertension, psychological stress, acute stress, chronic stress, bone density, osteoporosis, functional morphology

## Abstract

**Background/Objectives:** Chronic exposure to stress has been considered a risk factor for hypertension, which is also associated with increased bone resorption. This review aimed to investigate the effect of acute and chronic stress, associated with hypertension, on the skeletal system. **Methods:** A comprehensive search was conducted across multiple databases, focusing on peer-reviewed articles published in English. We include experimental, clinical, and peer-reviewed studies focused on the relationship between stress, hypertension, and bone resorption. Searches were conducted in MEDLINE via PubMed, Embase and Scopus, with the last search completed on 10 September 2024. **Results:** The main topics include situations that favor bone loss, such as psychological stress, which can lead to osteoporotic fractures through immunological and endocrine mechanisms. The relationship between psychological stress and loss of bone density, as in osteoporosis, occurs due to the reduction in the number of osteoblasts and loss in the balance between physiological formation/resorption. **Conclusions:** Chronic stress significantly affects cardiovascular health and bone resorption. This narrative review study highlights the vulnerability of the skeletal system, along with the cardiovascular system, to prolonged stress, emphasizing the need for multidisciplinary strategies in preventing stress-related conditions. Effective stress management can help reduce the risks of cardiovascular disease and bone resorption, emphasizing their role in comprehensive health care.

## 1. Introduction

Among cardiovascular diseases, hypertension is considered a serious and silent disease [1]. According to the World Health Organization (WHO), hypertension will be, among non-communicable diseases, the most important cause of functional disability in the coming decades. Lifestyle risk factors that contribute to the development of hypertension include sedentary lifestyle, smoking, diets rich in fat, and non-restorative nights of sleep [2]. Psychosocial factors may also play an important role in increasing blood pressure. Exposure to chronic stress has been identified as a risk factor. Occupational stress, stressful aspects of the social environment and low socioeconomic status have been studied [3].

The impact of stress on the development of hypertension is believed to involve a sympathetic nervous system response, in which the release of catecholamines leads to increased heart rate, cardiac output, and blood pressure [4]. The literature indicates that individuals with a greater reaction to stressful tasks are 21% more likely to have high blood pressure than those with a lower reaction, which suggests the importance of stress control in the non-drug treatment of high blood pressure [5]. In the literature, there are a few studies that show the effects of acute stressors, demonstrating the importance of different stress protocols, but continued exposure to stress may be more plausibly linked to sustained elevations in blood pressure and the incidence of hypertension [6].

Therefore, changes in lifestyle, especially the inclusion of physical exercise and its relationship with high blood pressure, require attention. In addition to being a notable but modifiable risk factor for stroke and coronary artery disease, high blood pressure also leads to atrial fibrillation, congestive heart failure and kidney failure, in addition to being an important component of metabolic syndrome [7]. The beneficial effect of regular exercise not only leads to a reduction in blood pressure, but it has also been shown to reduce left ventricular hypertrophy and improve quality of life. When combined with dietary changes, regular exercise causes reduced oxidative stress, increases nitric oxide availability, and improves overall metabolic profile [8].

As for bone tissue, under the influence of gravity, it undergoes greater or lesser deformation according to the intensity of daily life activities. It is known that activities that result in impact are those that most stimulate osteogenesis and thus reduce the loss of bone mass. Knowing how bone cells recognize the mechanical deformation imposed on the bone and initiate a series of biochemical chain reactions is of fundamental importance for the development of therapeutic and preventive practices [9]. Furthermore, evidence indicates that high blood pressure is associated with abnormalities in calcium metabolism: calcium loss can lead to increased bone mineral loss in individuals with high blood pressure [10]. Essential (or idiopathic or primary) hypertension, the most common type of hypertension, has different effects on bone mineral density in different locations [11].

With societal trends in aging (increased lifespans) and lifestyle (such as increased intake of high-fat foods and reduced physical activity), the prevalence rates of essential or primary hypertension and osteoporosis increase each year. Both hypertension and osteoporosis are age-related diseases and result from the interaction of genetic and environmental factors [12].

Furthermore, the prevalence of chronic disorders, high blood pressure and bone pathologies, as well as emotional disorders, such as stress, in the general population, creates a need for studies on these topics, with the aim of better understanding the physiological, morphological and biochemical aspects of and changes in these conditions. Therefore, investment in studies in this area that work towards new “multi-targeted” drug therapies, so that patients with this profile suffer fewer side effects. Considering these propositions and needs, this project aims to characterize the influence of varied acute and chronic stress associated with arterial hypertension on the skeletal system.

### Literature Search Method

A comprehensive search was performed using relevant keywords hypertension; psychological stress; acute stress; chronic stress; bone density; osteoporosis; functional morphology. Databases used for the search included MEDLINE (via PubMed), Scopus and Embase, with the last search completed on 10 September 2024. Subsequently, titles and abstracts were carefully screened to detect duplicates and further narrow the search. Two independent reviewers (J.P.M.I. and M.R.P.) conducted the searches, resolving any discrepancies through discussion and consensus. We included clinical, experimental and review studies, focusing on examining the association of stress with hypertension and bone resorption. Cohort, cross-sectional and interview studies were excluded from this review.

## 2. Bone Disorders

Bone is a dynamic tissue and is in a constant process of remodeling to maintain ideal mass and integrity throughout life [13]. It has basic functions such as support, protection and locomotion and is under the control of systemic factors, such as hormones, and local factors, such as growth factors and cytokines [14].

The homeostasis of the skeletal system depends on balanced bone remodeling, that is, on the balanced dynamics between the activity of osteoblasts (bone formation cells) and osteoclasts (bone resorption cells). In addition to osteoblasts and osteoclasts, bone tissue also contains cells called osteocytes [15]. Osteocytes are the most abundant cell type in bone tissue and are considered essential for bone maintenance as well as bone remodeling, since it has been suggested that osteocyte apoptosis can attract and stimulate osteoclast activity [16].

Bone is made up of an organic part (consisting of type I collagen, proteoglycans and adhesive glycoproteins) and an inorganic part (phosphate ions, calcium and, to a lesser extent, bicarbonate, magnesium, potassium, sodium and citrate) [17]. The union of phosphate and calcium forms crystals with a hydroxyapatite structure, typically associated with collagen fibers, that provides the resistance and hardness characteristics of bone tissue [18]. An important group of glycoproteins, extracted from the demineralized bone matrix, are bone morphogenetic proteins (BMPs), which are responsible for bone induction. Research shows that there are differences among the more than 20 BMPs discovered to date in their ability to induce osteogenesis [19]. BMPs 2, 6 and 9 appear to have greater potential to induce osteoblastic differentiation from mesenchymal progenitor cells [20].

When there is an imbalance in the activity of bone cells or matrix components associated with systemic and local factors, bone pathologies may arise [21]. Among the most common pathologies, osteoporosis, arthritis and osteosarcoma can be highlighted [22]. In the dental area, the presence of bone resorption associated with periodontal diseases, traumatic surgeries or even for physiological reasons due to lack of ridge function or inadequate prosthetic load is very common [23].

Thus, due to its importance in both the medical and dental fields, there is great development in approaches to bone disorders associated with other diseases, drug therapies, biomaterials, and grafts [24].

## 3. Emotional Stress

Stress is a set of organism reactions characterized by an imbalance in homeostasis in response to threats and/or aggressions of a psychic or physical nature, unusual or hostile, arising from environmental stimuli [25]. Stress designates all non-specific effects of factors that can act on the organism and has been characterized as a general adaptation syndrome, composed of three phases: alarm reaction, adaptation phase and exhaustion phase [26].

Stress comprises daily events that increase physiological activities and consequently cause psychological exhaustion to a certain extent. Modern life events, such as family and work-related problems, social withdrawal, financial concerns and violence, are some factors that can predispose or increase stress [27].

Psychological stress is characterized by a particular relationship between the individual and the environment, which is interpreted by the individual as a process of overload that exceeds their possibilities of adaptation and threatens their well-being. Stress situations promote a series of physiological and behavioral changes that can affect hormonal and neurotransmitter systems, causing different responses in the same individual to each new situation [28].

Animal models have been used to study the consequences of stress and its relationship with so-called stress-related disorders [29]. This is because absence of stress is a powerful indicator of the animal’s well-being. In rats, stress also modulates reproductive function [30]. The chronic stress protocol has been applied to animal models and is widely used to investigate the physiological and behavioral consequences of long-term stressful conditions [31]. It has been evidenced that the activity of the hypothalamic-pituitary adrenal (HPA) axis is directly linked to the body’s physiological responses to stress. Knowing that the HPA axis is formed by adrenocorticotropic hormones (ACTH) and corticosterone, serum concentrations of these hormones are considered an important indicator of stress, including in animal models [32].

## 4. Arterial Hypertension

Arterial hypertension (AH) is part of the group of cardiovascular diseases that represent the highest percentage of causes of mortality, such as cerebrovascular accident (CVA) and acute myocardial infarction [33]. This multifactorial clinical entity is characterized by the presence of systolic blood pressure (SBP) levels persistently equal to or above 140 mmHg and/or diastolic blood pressure (DBP) levels persistently equal to or above 90 mmHg [34].

In individuals without preexisting heart disease, the proportion of unrecognized myocardial infarctions increases significantly with the severity of hypertension. Thus, hypertensive patients have an increased risk of myocardial infarction and also of such an event not being recognized [35].

Arterial hypertension can be primary/essential or secondary [36]. The causes of primary arterial hypertension are not known in most cases, whereas secondary arterial hypertension must be investigated, since the etiological diagnosis means, in many cases, the possibility of specific treatment and cure or control by clinical or surgical intervention [37].

As for the known risk factors for hypertension, the most important are obesity, smoking, alcohol intake, family history of hypertension, psychological factors, certain personality traits and stress, which can be important triggers in the development of hypertension [38]. Genetic factors, as well as environmental factors such as physical inactivity and excessive sodium consumption, are also mentioned [39].

Arterial hypertension and osteoporosis are diseases considered predominant among the most prevalent chronic diseases in the elderly population [40]. Considering the rate of aging of the Brazilian population and the increase in the life expectancy of these elderly people, there is an increase in demand for specialized health services for this population and for studies that target bone and cardiovascular diseases.

Animal models using spontaneously hypertensive and normotensive rats have been used to study chronic conditions of arterial hypertension [41]. However, to date, few studies have used these models for investigating factors related to bone disorders and hypertension. Furthermore, reports on skeletal changes in hypertensive animal models are conflicting [42].

## 5. Relationship Between Emotional Stress and Bone Disorders

### 5.1. Emotional Factors

Emotional stress is closely related to abnormalities in bone metabolism and can lead to reduced bone mass and increased risk of fractures [43,44,45]. Bone metabolism is a physiological process regulated through the sympathetic nervous system by substances produced by the hypothalamus [46], as well as in the hypothalamus, where emotions are controlled [47]. Thus, changes in the central nervous system can induce changes in bone mass. The humoral mechanism initiated and orchestrated by the central nervous system has been perceived as a fundamental regulatory pathway between the nervous system and bone metabolism [48]. The regulatory pathway for bone metabolism arises from leptin. Leptin modulates bone mass, altering sympathetic tone after acting on the hypothalamus [49]. This causes peripheral nerves to release norepinephrine into the local microenvironment, which in turn activates β-adrenergic receptors expressed by osteoblasts [50]. Other molecules, such as neuromedin U, also regulate bone mass through signals processed in the hypothalamus. These signals, in turn, modulate sympathetic signals transmitted by peripheral nerves [51].

Intraosseous nerves are well distributed throughout the skeleton, including cortical and trabecular bone, bone marrow, and periosteum. Intraosseous motor nerves are mainly visceral motor nerves, which are divided into adrenergic and cholinergic nerves according to their released neurotransmitters. These peripheral nerves communicate with the skeleton to regulate bone metabolism through resident nerve cells, locally released neurotransmitters, neuropeptides, axon guidance factors, and neurotrophins [52]. Bone metabolism is regulated by signals generated by intraosseous nerves. These signals mediate bone mass and maintain bone micro- and macroarchitecture through regulation of bone deposition by osteoblasts and resorption by osteoclasts [53].

Depression is responsible for abnormal emotional and physical symptoms that include loss of interest and dysregulated sleep. Anxiety can be accompanied by physical symptoms, such as increased heart rate and shortness of breath. These mood disorders often have a common cause: psychological stress. In addition to osteoporosis, bone abnormalities related to psychological stress are regulated by peripheral nerves [54].

**Figure 1:**
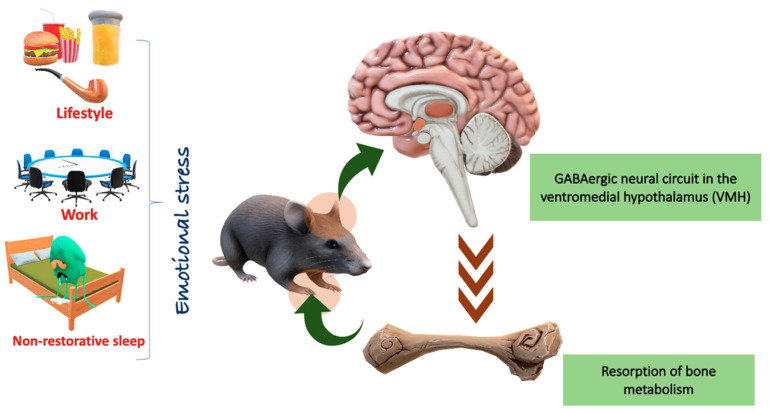
Illustrative diagram of the factors that cause emotional stress, such as a unhealthy lifestyle (food rich in sodium, fat and lack of nutrients), alcohol, cigarettes, excessive work, non-restorative nights of sleep and a sedentary lifestyle, which contributes to depression and anxiety (emotional control). These factors influence the GABAergic neural circuit in the ventromedial hypothalamus (VMH), influencing bone loss induced by chronic stress. Created by Biorender.com.

Psychological stress can accelerate bone loss and osteoporotic fractures through immunological and endocrine mechanisms. The relationship between psychological stress and osteoporosis has been demonstrated with chronic mild stress, an established model in rodents that leads to depression, causing a reduction in the number of osteoblasts, bone loss and reduced bone formation. Peripheral nerves participate in the regulation of bone metabolism in individuals with psychological stress. One way in which psychological stress may impact the risk and severity of osteoporotic disease is through catecholamine-induced activation of β -adrenergic receptors on osteoblasts and osteoclasts, which may increase RANKL expression and result in osteoclast differentiation [55] (Figure 1 and Table 1).

Chronic stress protocol has already been studied in both experimental and clinical study models. It was observed that there is a relationship between emotional stress and bone resorption (Table 1). In addition to the chronic stress protocol, there is also the possibility of occasional stress, which is called acute stress. It is interesting to be able to investigate the action of acute stress on bone resorption, which is not yet available in literature.

The relationship between bone tissue and emotional stress is a complex and growing field of study in medical research. Chronic stress leads to an increase in the production of hormones such as cortisol. High cortisol levels have been linked to reduced bone mineral density, which may increase the risk of osteoporosis and fractures [84]. Cortisol can interfere with bone formation and promote bone resorption, where bone tissue is destroyed faster than it is formed. Chronic stress leads to an increase in the production of hormones such as cortisol. Elevated cortisol levels have been linked to reduced bone mineral density, which may increase the risk of osteoporosis and fractures [85]. Cortisol can interfere with bone formation and promote bone resorption, where bone tissue is destroyed faster than it is formed. Stress can also increase inflammation in the body. Elevated inflammatory markers are associated with bone loss and can exacerbate conditions such as arthritis. People under stress can adopt behaviors that are harmful to bone health, such as an inadequate diet, a sedentary lifestyle or excessive consumption of alcohol and tobacco [86].

These behaviors can contribute to the deterioration of bone health. Mental health conditions such as depression and anxiety, often associated with stress, can also impact bone health. Depression, for example, can be associated with a decrease in physical activity and a poor diet, both factors that affect bone health. Research has shown that chronic stress can have an adverse effect on bone health over time. Longitudinal studies indicate that prolonged exposure to stress can accelerate bone loss and increase susceptibility to fractures. Interventions that reduce stress, such as stress management techniques, therapy, and wellness practices, can have a positive impact on bone health by helping to mitigate some of the adverse effects of chronic stress [87]. Emotional stress can negatively influence bone health through hormonal, behavioral and psychosocial mechanisms. It is important to consider integrated approaches to managing stress and maintaining bone health to promote overall well-being [88].

The relationship between bone, stress and inflammatory markers is an important aspect of bone biology and the stress response. Chronic stress can trigger an inflammatory response, which in turn can negatively impact bone health. Chronic stress can increase the production of inflammatory cytokines, such as TNF-alpha (tumor necrosis factor alpha), IL-1 (interleukin-1), IL-6 (interleukin-6) and CRP (C-reactive protein). These cytokines are involved in the inflammatory response and can influence bone health. Inflammatory cytokines, especially IL-1 and IL-6, can stimulate the activity of osteoclasts, the cells responsible for bone resorption. Increased osteoclast activity leads to greater degradation of bone tissue, contributing to the loss of bone mass [89]. Chronic inflammation can promote an environment in which bone remodeling processes are unbalanced, with a predominance of resorption over bone formation. Inflammatory cytokines can affect the bone matrix, the structure in which osteoblasts deposit new bone, interfering with mineralization and bone integrity.

Chronic stress is associated with an increase in the production of inflammatory markers, which in turn negatively affect bone health. Inflammatory cytokines can increase osteoclast activity and inhibit osteoblast function, resulting in bone loss. Furthermore, chronic inflammation can unbalance the bone remodeling process and compromise bone integrity. Therefore, managing stress and reducing inflammation are important for maintaining bone health and preventing related diseases [90].

### 5.2. Lifestyle

People under stress can adopt behaviors that are harmful to bone health, such as an inadequate diet, a sedentary lifestyle or excessive consumption of alcohol and tobacco. These behaviors can contribute to the deterioration of bone health. Bone loss is strongly influenced by different lifestyle habits. Daily habits can have a significant impact on bone health, affecting both bone formation and resorption. Calcium is essential for the formation and maintenance of bones. A diet low in calcium can lead to a decrease in bone density. Calcium-rich foods include dairy products, dark green leafy vegetables, and fortified foods. Vitamin D is crucial for calcium absorption. Vitamin D deficiencies can harm bone health. Sun exposure and foods fortified with vitamin D can help maintain adequate levels. High levels of caffeine and excessive alcohol consumption can interfere with calcium absorption and bone formation. Moderating intake of these items can help maintain bone health.

A diet low in nutrients essential for bone health, such as magnesium and vitamin K, can contribute to bone loss. Physical exercise, especially weightlifting and weight-bearing activities such as walking and running, stimulates bone formation and improves bone density. Lack of physical activity can lead to loss of bone mass. Resistance exercises, such as weight training, are particularly beneficial for bone health as they help to strengthen muscles and bones [91].

In addition, we can mention lifestyle behaviors such as smoking and alcohol consumption. Smoking is associated with lower bone density and an increased risk of fractures. Smoking can harm bone formation and reduce calcium absorption. Excessive alcohol consumption can interfere with bone formation and increase the risk of fractures [92]. Alcohol can also affect the absorption of nutrients essential for bone health. Sun exposure is important for the production of vitamin D, which is necessary for the absorption of calcium and the maintenance of bone health. Recommendations to promote bone health associated with lifestyle habits are: Maintain a Balanced Diet: Include foods rich in calcium and vitamin D and avoid excess caffeine and alcohol; Exercise Regularly: Practice physical activities that include weight-bearing and resistance exercises; No Smoking: Avoid smoking and minimize alcohol consumption; Monitor Health Conditions: Manage medical conditions that may impact bone health and discuss the effects of medications with a healthcare professional; Check Nutrient Levels: Check and maintain adequate levels of calcium and vitamin D through diet and sun exposure. In summary, bone loss can be influenced by a series of lifestyle habits. Adopting a healthy lifestyle, which includes a balanced diet, regular physical activity and avoiding harmful behaviors, can help preserve bone health and reduce the risk of conditions such as osteoporosis [93].

Mental health conditions such as depression and anxiety, often associated with stress, can also impact bone health. Depression, for example, can be associated with a decrease in physical activity and a poor diet, both factors that affect bone health. Depression and anxiety often lead to decreased physical activity and can lead to poor diet. A lack of essential nutrients, such as calcium and vitamin D, can compromise bone health. Some medications used to treat depression, such as tricyclic antidepressants and serotonin reuptake inhibitors (SSRIs), can have side effects that impact bone health, although this relationship can vary. It is important to monitor and discuss any potential impacts with a healthcare professional [94].

Anxiety often causes sleep disturbances, and a lack of quality sleep can negatively affect bone health. Sleep is important for bone regeneration and maintenance. Seeking treatment for depression and anxiety through therapy, medication and psychological support can help improve quality of life and minimize the impact on bone health. Maintaining a balanced diet rich in calcium and vitamin D, exercising regularly and adopting stress management strategies are essential for bone health. Having regular bone density screenings and discussing with healthcare professionals the influence of mental health conditions and medications on bone health can help with early detection and prevention of bone problems [95]. In summary, depression and anxiety can impact bone health in several ways, primarily through reduced physical activity, a poor diet, and hormonal and behavioral changes. Addressing these conditions holistically and maintaining a healthy lifestyle can help protect and improve bone health.

**Figure 2:**
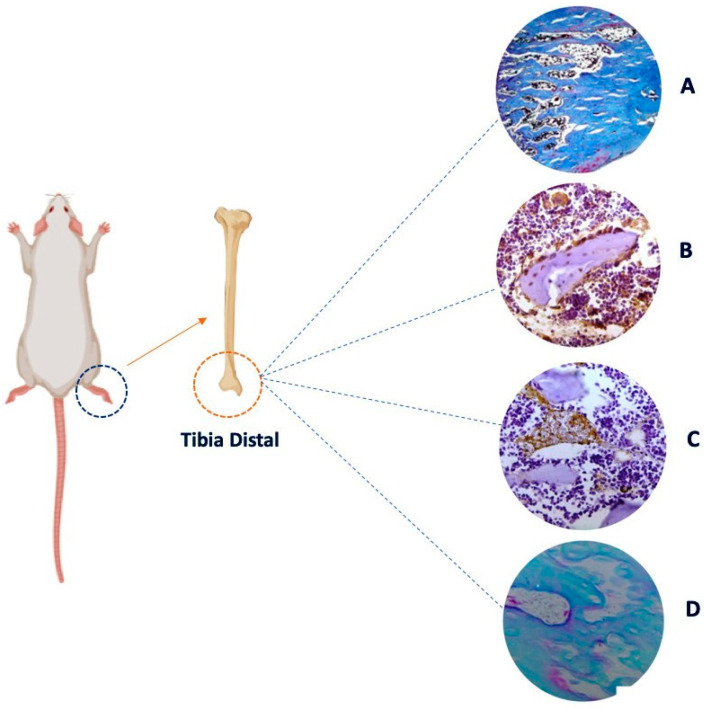
Representative images of a chronic stress protocol in rat tibia. (**A**) Bone tissue stained with Masson’s Trichrome. (**B**) Immunohistochemistry with osteocalcin that marks the presence of bone remodeling. (**C**) Immunohistochemistry with sialoprotein that marks osteoblastic activity. (**D**) Immunohistochemistry with tartrate-resistant acid phosphatase (TRAP) that marks osteoclast activity. 40× objective. Created by Biorender.com.

In summary, emotional stress can negatively influence bone health through hormonal, behavioral and psychosocial mechanisms. It is important to consider integrated approaches to managing stress and maintaining bone health to promote overall well-being (Figure 2).

## 6. Conclusions

The findings of this study reinforce the growing evidence that chronic stress plays a significant role not only in the etiology of cardiovascular diseases but also in bone health. Our observations indicate a clear association between chronic stress and bone resorption, suggesting that the skeletal system, like the cardiovascular system, is highly vulnerable to the deleterious effects of prolonged stress. This finding broadens our understanding of the systemic impact of stress, highlighting the need for a multidisciplinary approach in the prevention and treatment of stress-related conditions.

Moreover, the interrelationship between the cardiovascular and skeletal systems under stress underscores the importance of therapeutic interventions aimed at both reducing stress levels and protecting these systems. Effective stress management may be a crucial strategy for mitigating the risks of both cardiovascular diseases and bone resorption, preventing more severe complications in patients experiencing chronic stress.

The present study on the relationship between bone resorption and stress has some important limitations that must be considered when interpreting the results. The articles found in the literature mainly address chronic stress. It would be interesting to determine a study protocol with acute stress. Longitudinal and experimental studies will be needed to establish a more robust causal connection. Future studies could also explore the biological mechanisms underlying this interaction, including the action of stress hormones, such as cortisol, and their influence on osteoblasts and osteoclasts, in addition to evaluating the impact of different types and intensity of stress (acute and chronic), as well as possible therapeutic interventions to mitigate the negative effects of stress.

## Figures and Tables

**Table 1:** Relationship between emotional stress and bone resorption.

Author	Study Design	Sample Characteristics	Stress	Time	Main Effects
Ma et al., (2024) [56]	Review	ND	ND	ND	The autonomic neural basis of psychological stress-induced bone loss.
Pedreira et al., (2022) [57]	Review	ND	ND	ND	Transdermal estrogen therapy is a proven useful tool in women who do not respond to nutritional, psychological and/or modified exercise intervention and has beneficial effects on bone accumulation.
Indirli et al., (2022) [58]	Review	ND	ND	ND	Functional hypothalamic amenorrhea causes stress and bone is severely affected.
Liu et al., (2021) [59]	Experimental	Animal studies	Chronic	8 weeks	Glial-neuron microcircuit in VMH nuclei that mediates anxiety and bone loss induced by chronic stress.
Tkacheko et al., (2021) [60]	Clinical	Human studies	Chronic	ND	The additional involvement of the drug “Noofen^®^” in the complex of therapeutic measures allows stabilization of the psychological state, which indicates its effectiveness.
Ng & Chin, (2021) [61]	Review	ND	ND	ND	Chronic psychological stress should be recognized as a risk factor of osteoporosis.
Yang et al., (2020) [62]	Experimental	Animal studies	Chronic	8 weeks	Chronic stress in crewmembers resulted in decreased bone density.
Otto et al., (2020) [63]	Review	ND	ND	ND	The nervous system tightly modulates bone metabolism and regeneration.
Lopes Castro et al., (2020) [64]	Experimental	Animal studies	Chronic	30 days	Chronic stress induces oxidative blood imbalance, which can potentiate or generate morphological, structural and metabolic damage to the alveolar bone.
Liu et al., (2019) [65]	Clinical	Human studies	Chronic	8 weeks	Music therapy significantly alleviated clinical symptoms in patients with osteosarcoma.
Li et al., (2019) [66]	Experimental	Animal studies	Chronic	4 weeks	Psychological stress aggravates inflammation in periodontitis tissues and leads to further activation of the nuclear factor kappa-B (NF-κB) signaling pathway.
Gomes et al., (2019) [67]	Experimental	Animal studies	Chronic	56 days	Stress caused by fear modifies a periapical lesion, increasing the size of bone loss and increasing the number of inflammatory cells.
Follis et al., (2019) [68]	Clinical	Human studies	Chronic	6 years	High social stress was associated with decreased bone mineral density.
Haffner-Luntzer et al., (2019) [69]	Experimental	Animal studies	Chronic	19 days	Chronic psychosocial stress leads to an imbalanced immune response after fracture via β-AR signaling.
Stefanaki et al., (2018) [70]	Review	ND	ND	ND	Modern lifestyle, such as a sedentary lifestyle and being overweight, associated with depression, anxiety and stress, alters body composition, including causing damage to the bone structure.
Schmidt et al., (2017) [71]	Experimental	Animal studies	Chronic	2nd and 3rd trimester of pregnancy	The bone mineral density of an animal’s tail is closely correlated with prenatal stress in the third trimester.
Foertsch et al., (2017) [72]	Experimental	Animal studies	Chronic	19 days	Chronic psychosocial stress negatively impacts endochondral ossification in the growth plate, affecting both longitudinal and appositional bone growth.
Okbay Gunnes et al., (2017) [73]	Clinical	Human studies	Chronic	ND	Effect of stress caused by disordered eating habits harms bone remodeling.
Henneick et al., (2017) [74]	Experimental	Animal studies	Chronic	4 weeks	Bone loss during chronic stress is mediated through increased glucocorticoid signaling in osteoblasts (and osteocytes) and subsequent activation of osteoclasts.
Kumano, (2015) [75]	Review	ND	ND	ND	Osteoporosis causes anxiety, depression, loss of social roles and social isolation, which leads to stress.
Azuma et al., (2015) [76]	Review	ND	ND	ND	Chronic stress activates the HPA axis and increases inflammatory cytokines, eventually leading to bone loss, inhibiting bone formation and stimulating bone resorption.
Azuma et al., (2015) [77]	Clinical	Human studies	Chronic	4 weeks	The stress group showed an increase in serum corticosterone levels and increased bone resorption.
Nahrendorf & Swirski, (2015) [78]	Review	ND	ND	ND	Lifestyle (stress) changes the number of macrophages, diverting bone marrow production to the periphery.
Baldock et al., (2014) [79]	Experimental	Animal studies	Chronic	ND	Neuropeptide Y acts by inhibiting the production/release of norepinephrine and corticosterone, thus protecting bone from loss during chronic stress, which has therapeutic potential.
Erez et al., (2012) [80]	Clinical	Human studies	ND	ND	Supporting evidence for the existence of associations between mood variables and decreased bone mass.
Lv et al., (2012) [81]	Experimental	Animal studies	Chronic	1, 3 and 5 weeks	Psychological stress increased plasma hormone levels and indicated increased expression of IL-1β and TNF-α in the TMJ.
Seferos et al., (2010) [82]	Experimental	Animal studies	Chronic	137 days	The calcium content of the mandible and the ratio between calcium content and mandible volume was decreased
Patterson-Buckendahl et al., (2008) [83]	Experimental	Animal studies	Chronic	6 weeks	Osteocalcin levels were reduced indicating inhibition of bone formation

ND: Abbreviation indicating that the study did not define sample characteristics, type or duration of stress.

## Data Availability

Not applicable.
